# The Importance of Early Psychological Assessment for Differential Diagnosis and Detection of Comorbidity in Children With Autism Spectrum Disorder

**DOI:** 10.3389/fpsyt.2021.671744

**Published:** 2021-05-12

**Authors:** Harieta Manolova, Mihaela Hristova, Svetla Staykova

**Affiliations:** Department of Psychiatry and Medical Psychology, Medical University - Sofia, Sofia, Bulgaria

**Keywords:** ASD, differential diagnosis, comorbidity, cognitive and socio-communicative development, psychological assessment

## Introduction

This opinion article aims to synthesize 20 years of experience in the application of tools for assessing early psychological development, presented by clinical psychologists working in the field of child psychiatry in Bulgaria. The main focus relates to the role of psychological assessment for differential diagnosis of ASD and other neurodevelopmental disorders and its significance as a starting point for outlining both the individual developmental trajectory and potential concomitant psychopathological symptoms at a later age.

### Detailed Assessment of Psychological Development in the Early Years

In both schizophrenia and autism, the principles of diagnostic classification evolve from “fragmentation” to “unification” of conditions within a common disorder (1). This trend emphasizes the necessity of conducting a comorbidity assessment. If we accept a definition of comorbidity as “the co-occurence of more than one disorder in the same individual” (2), the most common comorbid conditions in ASD include: intellectual disability, attention-deficit/hyperactivity disorder, language disorders, genetic disorders, etc. Additional co-occurring difficulties are often reported, i.e., sleeping, eating and elimination problems, physical and sensory problems, emotional and behavioral difficulties, etc. (3). The fact that children are affected to a different degree by the disorder additionally supports the hypothesis for the existence of specific biogenetic factors, whose configuration and number are probably related to the variations in severity (4). The presence of additional internal (including comorbid conditions) and external (environment-related) factors are believed to further aggravate the disorder (5). The typical “disharmony” detected in the psychological profiles of autistic children, which is believed to be more evident in severe cases, is indicative of the reported unevenness of development across spheres, as well as of the high internal inconsistency of personal achievements across the different dimensions measured. Bernard et al. (6) emphasize that developmental heterogeneity of children with autism is predominantly an aggravating factor for the severity of ASD. A high correlation between heterogeneity and severity in autistic subjects has been identified—the higher the heterogeneity, the lower the developmental level. Such findings in the course of assessment outline an individual trajectory of psychological development in all cases, leading to the elaboration of individualized intervention programs in the early years.

The assessment tools utilized by the authors are based on the hierarchical model described in Piaget's sensorimotor stage (7, 8). This approach allows “fragmentation” of the different achievements, expected within the six substages and thus makes it possible for the individualized recommendations to be inspired by the tools themselves. Comparison of profiles of psychological functioning of autistic children and children with other neurodevelopmental disorders in early childhood (1, 9–12), reveals interesting findings in the dimensional analysis of individual and intragroup relations between the studied developmental indicators. Typical characteristics of autistic cognitive, socio-communicative, socio-emotional and sensorimotor functioning were outlined, different from those of the normative group of healthy children, children raised in a situation of social and emotional deprivation (10) and children with Down syndrome (12). Clinical practice shows that such a thorough psychological assessment: contributes to early diagnosis, informs the elaboration of effective, individualized intervention programs and outlines clinical subgroups associated with different severity of the disorder.

### Assessment Tools and Specifications in the Profiles of Children With ASD

Recent developments in the fields of child psychiatry and clinical psychology, have posited a requirement for early detection of ASD aimed at preventing its aggravation. Clinical practice reveals the importance of working with parents as well as the positive effect of early therapeutic interventions on child development. The experience of interinstitutional work on individual cases executed by medical, educational and social services validates the adoption of a comprehensive approach to caring for children with autism. The interuniversity collaboration of research teams of clinical psychologists from Medical University - Sofia headed by H. Manolova and the Catholic University of Louvain-Belgium headed by N. Nader-Grosbois (2000–2002), led to the inauguration in practice of an original, comprehensive toolkit for early developmental assessment, that includes four scales briefly described below:

1. “*Scales for Assessment of Early Cognitive Development*” adapted for Bulgaria by Manolova et al. (11)—source: “Echelles d'Evaluation du Developpement Cognitif Precoce” (EEDCP), Nader-Grosbois (13), a revised version of the “Infant Psychological Developmental Scales” (IPDS), Uzgiris and Hunt (8).

This instrument contains seven scales corresponding to the six areas of cognitive development during the sensorimotor stage: visual follow up and permanence of object, means of attainment of wanted event, vocal imitation and gesture imitation, development of operational causality, space relations between objects, development of schemes between objects. Individual results are presented for every sensorimotor substage and each score corresponds to an estimated developmental age in months (14). Based on these results it is possible to create an individual profile of cognitive development and to assess the mean age of cognitive functioning (11, 12).

2. “*Scales for Early Assessment of Socio-Communicative Development*” adapted for Bulgaria by Manolova et al. (11)—source: “Early Social Communication Scales” (ESCS), Siebert and Hogan (15); revised version and verification of Guidetti and Tourette (16), with the participation of J.-L. Adrien. Protocols: Nader-Grosbois (13).

This instrument consists of eight scales related to three communication functions (social interaction, joint attention, and behavior regulation) and three communication roles (response, initiation, and maintenance). Performance is assessed according to the following combinations of communicative functions and communicative roles.

Results are presented as an estimated level of development (0 - Reflex, 1 - Simple, 2 - Coordinated, 3 - Conventional-Gesture, 3.5 - Conventional-Verbal (one word); 4 - Symbol (two words), as individual score and as age intervals of development (0–2, 3–7, 8–13, 14–21, over 22 months). Based on these results it is possible to create an individual profile of communicative development as well as to calculate the mean age of socio-communicative development (11, 12).

Research findings reveal a high correlation between the two scales. The application of both instruments combined has been well-researched and adapted to the assessment of children with ASD and other developmental conditions (13, 17). Disposing of reliable tools for assessing main developmental spheres in young children with autism contributes to the early identification and diagnosis. Furthermore, presenting results with a focus on capabilities rather than deficiencies makes it possible to avoid both labeling children and entering guilt-based interactions with parents.

In addition to the basic assessment of a child's cognitive and socio-communicative development, it is possible to explore the psychomotor capacity and some socio-emotional indicators-source: “Transdisciplinary Play-Based Assessment,” Linder (18), modified version Nader-Grosbois et al. (9).

3. “*Scale for Assessment of Sensorimotor Development*,” adapted for Bulgaria by Manolova et al. (11) contains the following five categories: a general aspect of the movements, tonus, strength and endurance, reactivity to sensory stimulation, postures and displacements, seizing, and manipulation.
4. “*Scale for Assessment of Socio-emotional Development*,” adapted for Bulgaria by Manolova et al. (11) contains seven categories: temperament, motivation to learn, interactions with a close adult, interactions with the examiner, peer interactions, nuances of interactions, mood.

These scales assess the level of adaptation of motor and emotional responses by the following three-tier criteria: adapted, moderately adapted, unadapted. The study of socio-emotional indicators in interactions with different people aids the understanding of communication dynamics in autistic children as influenced by different emotional triggers.

Over the years, we have gained extensive experience in the utilization of these tools for assessing children with neurodevelopmental disorders, supported by research (9, 12). This made it possible to outline specific functional characteristics and features in the profiles of children from various diagnostic groups. Based on the accumulated clinical and research practice, certain typical characteristics in autistic children's developmental profiles have been outlined. Some of these characteristics have been identified by other researchers as well (6, 13, 19, 20):

- disharmonious (inhomogeneous) profile of cognitive functioning;
- greater delay within the cognitive scales: “vocal imitation,” “gesture imitation” and “object permanence” (only in severe cases);
- greater overall delay in the socio-communicative sphere as compared to the cognitive;
- greater delay in “initiation” than in “response” and “maintenance” within the socio-communicative profile;
- higher results on “behavior regulation” as compared to other communication areas;
- higher performance on items that can be completed through a trial and error approach, as compared to items that require direct interaction with an adult.

The tools presented above allow for the use of materials that children with ASD often carry with them and consider valuable. This facilitates their inclusion in the assessment process in a natural manner. In addition, the flexible number of sessions (usually between 2 and 4) makes it possible for children to gradually adapt to the context, helps the clinician monitor progress and exerts a therapeutic effect on both children and their parents.

**Figure 1:**
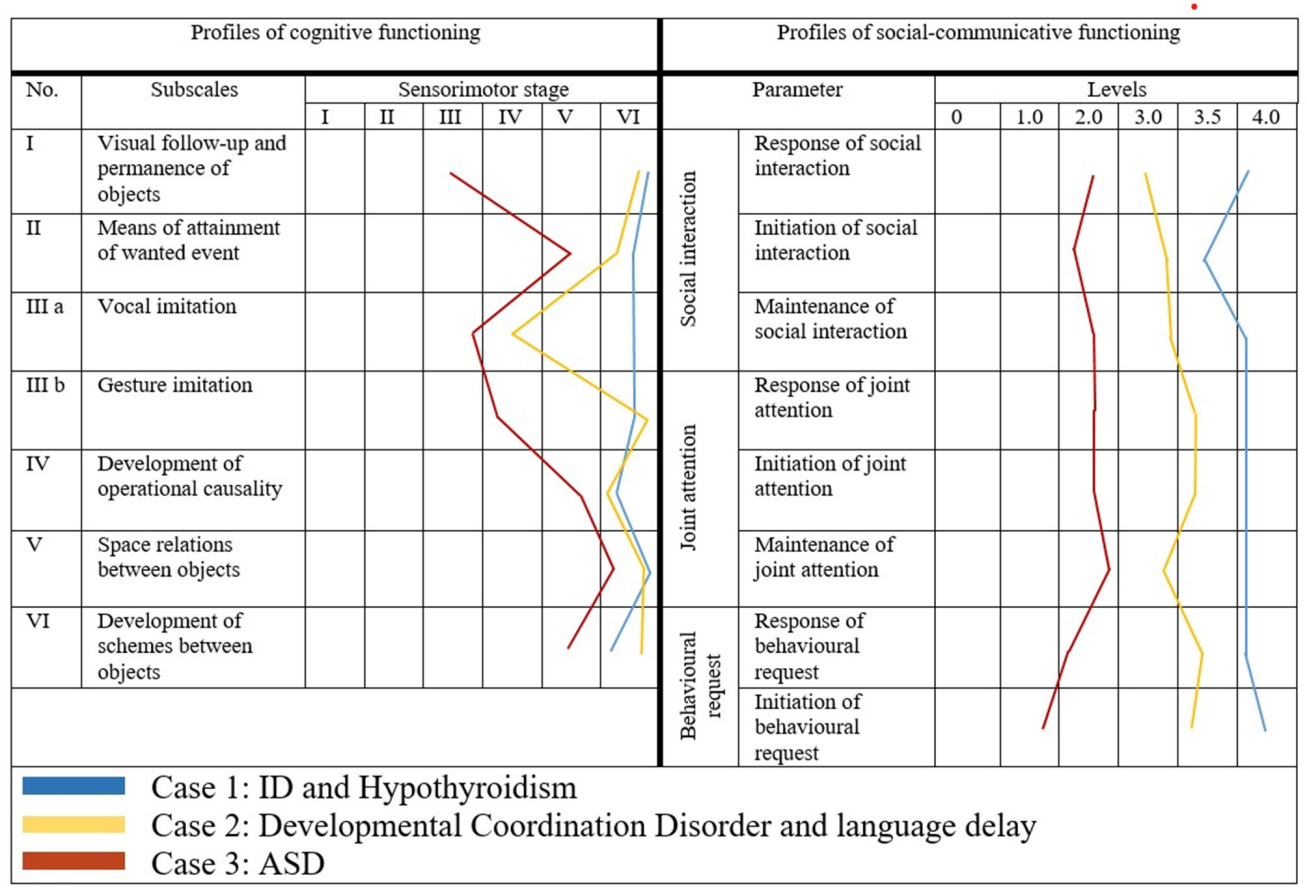
Profiles of psychological development of children with ASD and other neurodevelopmental disorders.

Figure 1 presents the cognitive and socio-communicative profiles of three cases as an illustration of the role of psychological assessment for diagnosis elaboration and detection of psychiatric comorbidity.

*Case one* is of a 42-month old boy, diagnosed with ASD outside Bulgaria (in the place of residence). The cognitive profile is homogeneous and reveals overall and significant delay in all substages of the sensorimotor period. The child is in a symbiotic relationship with his mother and displayed excessively negative response upon socialization in kindergarden where a language different form his mother tongue was spoken. He was aggressive toward the children and obsessed with two of them, whom he continuously imitated and even persecuted. At the age of 2½ years the child was diagnosed with Hypothyroidism and is eversince on thyroid medication. The results from the psychological assessment support a diagnosis of Intellectual Disability (ID) with comorbid anxiety and separation fears.

*Case two* is of a 44-month old girl with indications of being on the autism spectrum. The comprehensive psychological assessment did not support such a hypothesis, as she was actively using gestures and facial expressions to convey emotions, engaged in situations of joint attention and shared enjoyment and profiles on the various scales were homogeneous (except of “verbal imitation”). Primary motor underdevelopment was detected, affecting also the vocal apparatus (biting and chewing began only recently). The girl was diagnosed with a Developmental Coordination Disorder and was referred for speech therapy.

*Case three* is of a 41-month old boy and illustrates a typical profile of psychological development for a child with ASD. The cognitive profile is highly heterogeneous, with greater delay on “object permanence” and imitation scales. The overall socio-communicative development is significantly lower than cognitive development. The socio-communicative profile is dominated by a greater delay on “initiation” which additionaly supports the diagnosis of ASD. Despite its young age, there are indications of possible comorbidity with ADHD in the future.

## Discussion

Our research on existing international practices in the field of early psychological development and analysis of a series of papers on the topic (21–26), shows an increased interest in the early developmental period on behalf of clinicians and researchers. There is a tendency to decrease the age of ASD diagnosis in order to stimulate development and prevent the aggravation of autistic behaviors in the future. Our understanding of early psychological development is based on its dynamic, sequential and hierarchical nature, as well as on the interrelation between the various developmental spheres and with the relevant factors (biological, psychological, and social), which positively affect the dynamics of this process.

The application of tools for a detailed assessment of psychological functioning in the early years makes it possible to elaborate early intervention programs aimed at optimal compensation for potential underdevelopment or deviations in various developmental aspects. Such interventions can start even before a child receives a diagnosis, as early as 18 months. In this regard, it is very important to have a comprehensive and efficient toolkit for early developmental assessment, that takes into account the importance of social interactions and emotional relationships. A toolkit that objectifies results across domains and allows for comparison of individual achievements both against the statistically determined norms and against individual results, rhythm, and course of development.

Early assessment of development can also serve as an important basis for follow-up assessment and treatment. Individual profile characteristics and observation of early childhood behaviors can be indicative for potential comorbidity and/or developmental abnormalities (27, 28) that can be detected later in life. ASD is often accompanied by cognitive deficits and speech delay (29), as well as by emotional and behavioral difficulties (30, 31). That is why psychological assessment is so important for both the differential diagnosis and the detection of comorbid conditions. For example, an experienced psychologist can distinguish symptoms that are due to the presence of severe intellectual deficits, rather than being signs of autism (especially in early childhood). Similar is the differentiation between symptoms resulting from systemic emotional and social deprivation and similar signs of diminished emotional reciprocity and social responsiveness as displayed by children with ASD.

An important feature of developmental profiles of young children with ASD is the significant disharmony (6, 13, 19, 20)—both across the different spheres of development and within the individual profile. It even seems as if there are “gaps” of undeveloped abilities existing together with the presence of abilities that are hierarchically more complex and “step” on something missing. This phenomenon, known as “islets” (1, 32, 33), has been well-documented by psychologists in the assessment of children on the spectrum. The presence of already formed skills and such in the process of acquisition, serve as a solid foundation of every effective therapeutic program. The most important starting point in the work of a clinical psychologist is to reveal what the child can do, instead of emphasizing things that he/she cannot do. While a child psychiatrist focuses on deficits and symptoms that are relevant for assigning an ASD diagnosis, a clinical psychologist is expected to highlight existing strengths that can inform the elaboration of effective interventions. Psychological assessment is the foundation on which therapeutic strategies are built to make the necessary recommendations and to identify feasible strategies for overcoming deficits.

An indisputable fact, reported by research and in clinical practice (1, 3, 9, 27), is that individualized interventions are of crucial importance for the improvement of many aspects, such as communication skills (including speech as an important predictor of better prognosis), cognitive capacity, etc. In addition, such interventions contribute to reducing the severity of symptoms and the level of stress in children, caregivers and their extended family. This opinion article presents an assessment model that makes it possible to detect autistic symptoms, as well as to outline an individual developmental trajectory and to identify possible comorbid conditions.

## Author Contributions

All authors listed have made a substantial, direct and intellectual contribution to the work, and approved it for publication.

## Conflict of Interest

The authors declare that the research was conducted in the absence of any commercial or financial relationships that could be construed as a potential conflict of interest.
